# Albendazole-induced granulomatous hepatitis: a case report

**DOI:** 10.1186/1752-1947-7-201

**Published:** 2013-07-26

**Authors:** Juan Ignacio Marin Zuluaga, Andres Eduardo Marin Castro, Juan Camilo Perez Cadavid, Juan Carlos Restrepo Gutierrez

**Affiliations:** 1 Hepatology and Liver Transplantation Unit, Hospital Pablo Tobon Uribe, Calle 78B No. 69-240, Medellin, Colombia

**Keywords:** Albendazole, Drug-induced liver injury, Granulomatous hepatitis, Idiosyncratic hepatotoxicity

## Abstract

**Introduction:**

Drug-related hepatotoxicity is a common medical problem with implications for health systems. It constitutes a cause of acute liver failure and, in many cases, is responsible for the rejection of new pharmacological agents during efficacy and safety studies. Risk factors, as well as pathogenesis of drug-induced liver injury, are poorly understood. The diagnosis of drug-induced liver injury is challenging; it is difficult to define the cause of drug hepatotoxicity due to the heterogeneity of the clinical presentation and the absence of established criteria for accurate and reproducible identification of drug-associated liver toxicity.

**Case presentation:**

We report the case of a 25-year-old Hispanic woman admitted to our Clinical Hepatology Unit with symptoms of acute hepatitis of unknown etiology. She was diagnosed with albendazole-induced granulomatous hepatitis after ruling out other possible causes, based on laboratory studies, liver biopsy, medical history, detailed drug history, and spontaneous improvement of her liver biochemical profile after medication withdrawal. This diagnosis was supported by the Council for International Organizations of Medical Sciences-Roussel Uclaf Causality Assessment Method, which showed a likely correlation between hepatocellular damage and drug toxicity as the etiology.

**Conclusions:**

Our patient’s suspected diagnosis was albendazole-induced granulomatous hepatitis with confirmatory histologic pattern. This case deserves particular attention due to the wide use of albendazole in our country (Colombia) and the prevalent medical issue of drug-related hepatotoxicity.

## Introduction

Granulomatous hepatitis is a difficult disease to diagnose. The etiology of these lesions is rarely determined histologically, and the presence of granulomas in the liver does not necessarily imply a granulomatous process. The lesion pattern is a granulomatous inflammatory reaction with the formation of chronic granulomas, composed of aggregates of cells, epithelioid macrophages arranged in a collar surrounded by mononuclear cells and occasional plasma cells. The etiology is usually infectious (tuberculosis, brucellosis, fungal or parasitic), but may also develop in autoimmune disease, such as primary biliary cirrhosis and rheumatic fever, as well as having an unknown origin, such as sarcoidosis, or foreign body reaction in response to drug-induced toxicity [1,2].

In order for a drug to be responsible for granulomatous hepatitis and make a definitive diagnosis of drug-induced liver injury (DILI), it is necessary to exclude other causes and demonstrate the improvement of the condition after stopping the offending drug [1]. Histologically, drug-induced hepatic granulomas are indistinguishable from those produced by other causes [3].

Patients usually present with symptoms such as fever, arthralgia, nausea, and vomiting in the month following drug initiation, although the symptoms may be delayed. Physical examination shows the presence of jaundice and hepatomegaly. Liver function tests show abnormalities of transaminases and alkaline phosphatase. The presence of hyperbilirubinemia and peripheral blood eosinophilia are not seen in all patients but, when present, suggest drug-induced hepatitis [1].

There are no absolute criteria or specific method for the diagnosis of toxic hepatitis. The most widely used scale is the Council for International Organizations of Medical Sciences (CIOMS)-Roussel Uclaf Causality Assessment Method (RUCAM), developed in 1995, which relates a causative agent and toxic liver damage, and has the following results: “highly probable”, “probable”, “possible”, “unlikely”, or “excluded”, according to the total score. This method has proven to be more accurate and reproducible compared with other methods published in the literature [4-6].

This article describes the case of a patient with albendazole-induced granulomatous hepatitis with confirmatory histologic pattern. This case deserves special attention due to the wide use of albendazole for the treatment of *Ascaris lumbricoides* and other helminth infections, sometimes without medical prescription in our country (Colombia). In the literature, there are few reports of granulomatous hepatitis associated with this group of drugs.

## Case presentation

This case report describes a 25-year-old Hispanic woman, without significant past medical history, who presented with progressive jaundice, associated with right upper quadrant pain, fatigue, weakness, dark urine, without acholia, fever, or vomiting. On physical examination, the patient had icteric sclerae, no hepatomegaly and no stigmata of chronic liver disease. The laboratory results are shown in Table 1. The results of ultrasonography of the liver and biliary tract were normal.

**Table 1:** Laboratory tests

**Test**	**Baseline**	**At 2 months**	**At 6 months**
Hemoglobin (g/dL)	12.7	12.0	13.0
Hematocrit (%)	44	43	42
Leukocytes (cells/mm^3^)	8.400	7.650	7.990
Blood Platelets (platelets/mm^3^)	140.000	250.000	180.000
Eosinophils (eos/mm^3^)	260	300	310
Total bilirubin (mg/dL)	13.75	1.1	1.2
Direct bilirubin (mg/dL)	13.66	0.4	0.6
Aspartate aminotransferase (U/L)	933	22	30
Alanine aminotransferase (U/L)	1649	23	31
Alkaline phosphatase (U/L)	145	75	62
Gamma glutamyl transpeptidase (U/L)	28	17	15
Hepatitis A immunoglobulin M antibodies	Negative		
Hepatitis B surface antigen	Negative		
Hepatitis C antibody	Negative		
Human immunodeficiency virus antibodies	Negative		
Anti-smooth muscle antibodies	Positive 1:40	Negative	
Antinuclear antibodies	Negative	Negative	
Immunoglobulin G (mg/dL)	1.300	1.260	
Cytomegalovirus immunoglobulin M	Negative		
Epstein–Barr immunoglobulin M	Negative		
Herpes simplex immunoglobulin M	Negative		
Varicella immunoglobulin M	Negative		

**Figure 1:**
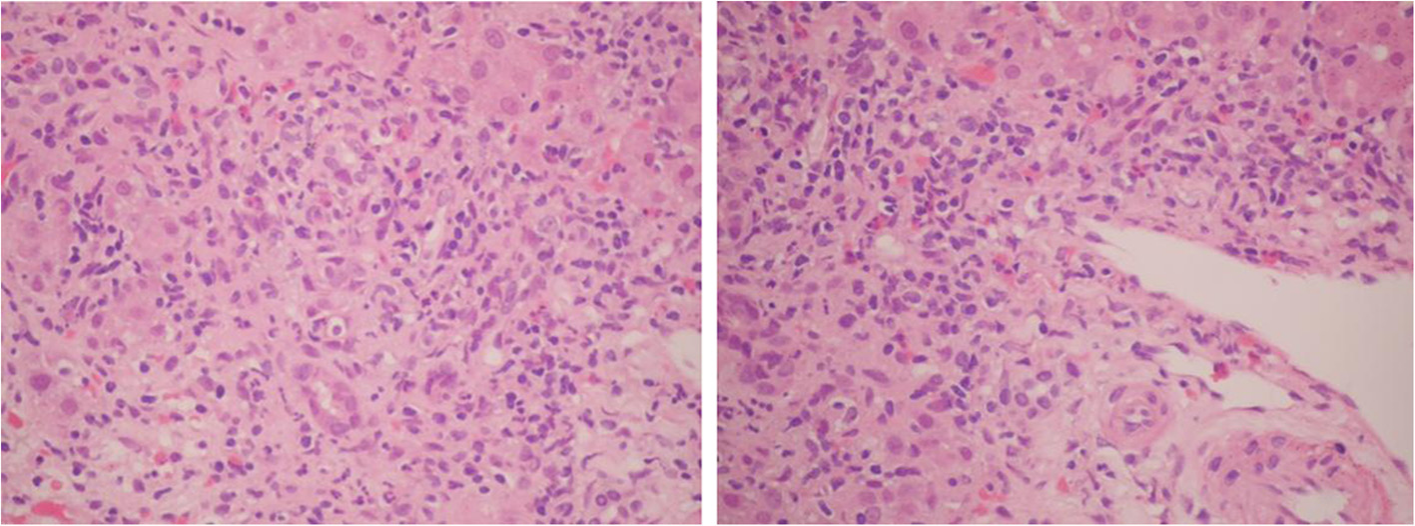
**Hematoxylin and eosin stain.** Portal tracts infiltrate with moderate and mixed inflammation, consisting of lymphocytes, plasma cells, neutrophils, and eosinophils with moderate interface activity. Inflammatory Activity Grade 3 to 4.

**Figure 2:**
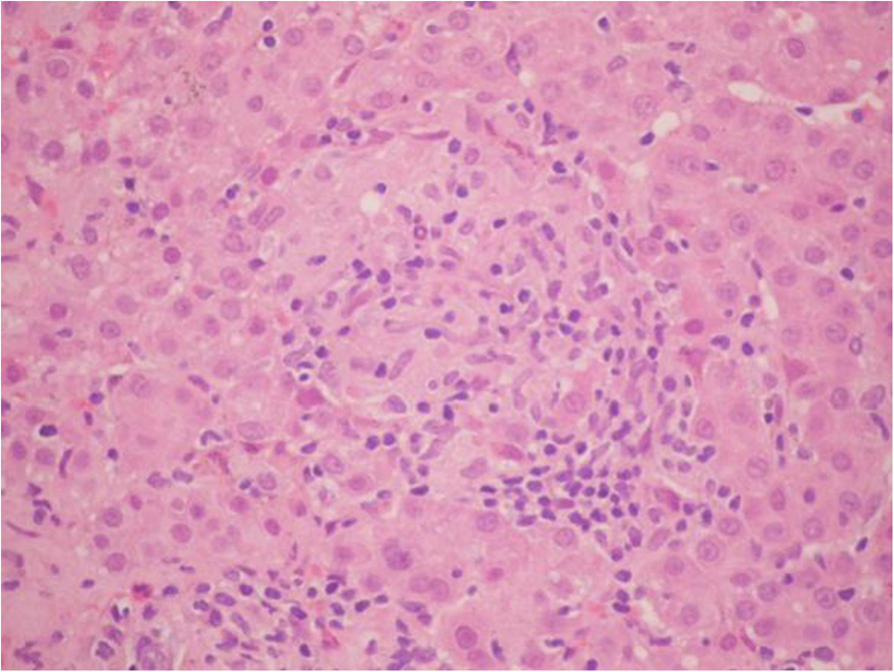
**Hematoxylin and eosin stain.** Hepatic parenchyma with presence of epithelioid macrophages that have formed granulomas without necrosis, interspersed with lymphocytes. Inflammatory activity around the granulomas is characterized by inflammatory lobular cells between the sinusoids and hepatocytes.

With the clinical and laboratory diagnosis, a preliminary diagnostic approach to acute hepatitis of unclarified etiology was established. We performed a percutaneous liver biopsy guided by ultrasound, which showed portal inflammatory infiltrate of lymphocytes, plasma cells and neutrophils, with necrosis of the hepatocytes of the limiting plate, and macrophage infiltrate composed of epithelioid granulomas, forming a non-necrotizing aspect in the hepatic sinusoids (Figures 1 and 2). Special stains for tuberculosis and fungi were negative. Contrasted computed tomography of the chest and abdomen were normal, ruling out sarcoidosis. The patient had a favorable outcome, with gradual improvement of her liver biochemical profile, without any specific treatment. At this point, we considered the possibility of drug-related granulomatous hepatitis. A more detailed questioning revealed that 2 weeks before the jaundice, the patient had received empirical treatment with albendazole and paracetamol and hyoscine butyl bromide for nonspecific gastrointestinal symptoms. The stool specimen was normal.

The self-limiting condition, as well as the gradual normalization in her liver profile, supported the possibility of toxic hepatitis. Patient monitoring without any intervention was decided. Table 1 shows some of the follow-up analysis after 2 and 6 months. The results demonstrated spontaneous resolution of toxic granulomatous hepatitis.

## Discussion

Drug-related hepatotoxicity is a common medical problem, with implications for health systems, and is also a cause of acute liver failure and, in many cases, responsible for the elimination of new pharmacological agents during efficacy and safety studies. Risk factors and pathogenesis of DILI are poorly understood. The diagnosis of DILI is one of exclusion, because it is not easy to define a toxic etiology as the cause of liver disease due to the heterogeneity of the clinical presentation and evolution. It can range from transient elevations in liver enzymes to liver failure and even symptomatic chronic liver disease [3,7].

Studies indicate that antibiotics, agents with central nervous system action, herbal supplements, and immunomodulatory agents are the most common cause of DILI in the United States of America. Hepatocellular injury patterns are more common in younger patients, whereas patterns of cholestasis prevail in older patients [3,5,7]. These findings are consistent with those found in other studies where antibiotics, nonsteroidal anti-inflammatory drugs, and immunomodulatory drugs have been implicated as a cause of drug-induced hepatotoxicity [6,8-10]. However, acute liver failure is rare, and up to 17% of cases are attributed to idiosyncratic reactions, determined by individual susceptibility and not related to the dose of the drug. There are no models for predicting adverse reactions [9,11].

The Naranjo Probability Scale and Clinical Diagnostic Scale are also used to assess the causality of drug-induced hepatotoxicity [6]. The CIOMS-RUCAM scale showed a “possible” relationship with a score of five points, which supported the diagnosis of albendazole-induced granulomatous hepatitis with a pattern of hepatocellular DILI damage. The clinical and biochemical improvement of the patient after stopping the medication, without recurrence, confirms the diagnosis [4-6].

In clinical trials, treatment with albendazole has been associated with mild to moderate elevations of liver enzymes in about 16% of patients. Although generally returned to normal after discontinuation of treatment, there have been reports of persistent hepatitis [12].

At the time of this review, only three articles were indexed in MEDLINE: the first describes a case of granulomatous hepatitis induced by mebendazole administration in a patient with cytolytic hepatitis with eosinophilia to treat infestation *Ascaris* in the presence of fever, diarrhea, and eosinophilia. Liver biopsy results were consistent with the diagnosis of granulomatous hepatitis [2]. Another report in the literature describes the case of a patient who had consumed albendazole 6 hours earlier, and for whom alterations in liver function tests, necrosis, and steatosis characteristics of hepatocellular hepatitis were documented [12]. The diagnosis of drug-induced hepatitis or DILI was based also on the evaluation method CIOMS-RUCAM, with a score of nine (“highly probable”) [4]. The exclusion of other possible causes and spontaneous liver profile improvement also supported this diagnosis.

The third case reported acute hepatitis with jaundice and slightly elevated liver function tests in a 7-year-old patient after administration of oral albendazole [13]. This side effect has been known after prolonged administration, but as far as it is known, acute hepatitis has never been described in infants.

Compared to the case presented in the reports mentioned above, the diagnosis in these reports was conducted by history of ingestion of an antiparasitic agent and the presence of eosinophilia, the difference with the case presented relied on the presence of fever and the period of time until symptoms developed.

The liver abnormalities are classified into three types: hepatocellular, cholestatic, and combined, according to the levels of liver enzymes [12]. In the reports mentioned above, there were established hepatocellular damage patterns and granulomatous reactions, as in the case presented. In our case, the positivity of anti-smooth muscle antibodies could be explained by a false positive due to hepatocellular necrosis. A subsequent review of all autoimmune markers indicated that these were negative. The clinical presentation of this case is unspecific, but it is consistent with other reported cases of DILI [2,12,13].

In many countries, the health system must be advised of the importance of controlling the sale of “over-the-counter” drugs, including antibiotics, in order to avoid unnecessary hepatotoxicity.

## Conclusions

This case illustrates how albendazole-induced granulomatous hepatitis, although rare, should be considered in patients in whom other causes have been excluded, thus presenting resolution of symptoms after medication withdrawal. The initial clinical presentation of this patient, laboratory findings, previous use of albendazole, and self-limiting condition with a gradual normalization of liver profile after removal of the drug, supported the possibility of toxic hepatitis. The diagnosis of DILI is one of exclusion, because it is not easy to define a toxic etiology as the cause of liver disease due to the heterogeneity of the clinical presentation and evolution. The CIOMS-RUCAM scale showed a “possible” relationship with a score of five, which supported the diagnosis of albendazole-induced granulomatous hepatitis with a pattern of hepatocellular damage. This case deserves special attention due to the wide use of albendazole in our country and because drug-related hepatotoxicity is a frequent medical problem.

## Consent

Written informed consent was obtained from the patient for publication of this case report and any accompanying images. A copy of the written consent is available for review by the Editor-in-Chief of this journal.

## Competing interest

The authors declare that they have no conflicts of interest.

## Authors’ contributions

JIM, AEM, and JCR analyzed and interpreted the patient’s data regarding liver studies. JCP analyzed and interpreted the patient data regarding the performed liver studies and liver histology. All authors wrote, read, and approved the final manuscript.
